# Factors that contribute to long-term survival in patients with leukemia not in remission at allogeneic hematopoietic cell transplantation

**DOI:** 10.1186/1756-9966-30-36

**Published:** 2011-04-10

**Authors:** Hideo Koh, Hirohisa Nakamae, Kiyoyuki Hagihara, Takahiko Nakane, Masahiro Manabe, Yoshiki Hayashi, Mitsutaka Nishimoto, Yukari Umemoto, Mika Nakamae, Asao Hirose, Eri Inoue, Atsushi Inoue, Masahiro Yoshida, Masato Bingo, Hiroshi Okamura, Ran Aimoto, Mizuki Aimoto, Yoshiki Terada, Ki-Ryang Koh, Takahisa Yamane, Masahiko Ohsawa, Masayuki Hino

**Affiliations:** 1 Hematology, Graduate School of Medicine, Osaka City University, Osaka, Japan; 2 Diagnostic Pathology, Graduate School of Medicine, Osaka City University, Osaka, Japan

## Abstract

**Background:**

There has been insufficient examination of the factors affecting long-term survival of more than 5 years in patients with leukemia that is not in remission at transplantation.

**Method:**

We retrospectively analyzed leukemia not in remission at allogeneic hematopoietic cell transplantation (allo-HCT) performed at our institution between January 1999 and July 2009. Forty-two patients with a median age of 39 years received intensified conditioning (n = 9), standard (n = 12) or reduced-intensity conditioning (n = 21) for allo-HCT. Fourteen patients received individual chemotherapy for cytoreduction during the three weeks prior to reduced-intensity conditioning. Diagnoses comprised acute leukemia (n = 29), chronic myeloid leukemia-accelerated phase (n = 2), myelodysplastic syndrome/acute myeloid leukemia (MDS/AML) (n = 10) and plasma cell leukemia (n = 1). In those with acute leukemia, cytogenetic abnormalities were intermediate (44%) or poor (56%). The median number of blast cells in bone marrow (BM) was 26.0% (range; 0.2-100) before the start of chemotherapy for allo-HCT. Six patients had leukemic involvement of the central nervous system. Stem cell sources were related BM (7%), related peripheral blood (31%), unrelated BM (48%) and unrelated cord blood (CB) (14%).

**Results:**

Engraftment was achieved in 33 (79%) of 42 patients. Median time to engraftment was 17 days (range: 9-32). At five years, the cumulative probabilities of acute graft-versus-host disease (GVHD) and chronic GVHD were 63% and 37%, respectively. With a median follow-up of 85 months for surviving patients, the five-year Kaplan-Meier estimates of leukemia-free survival rate and overall survival (OS) were 17% and 19%, respectively. At five years, the cumulative probability of non-relapse mortality was 38%. In the univariable analyses of the influence of pre-transplant variables on OS, poor-risk cytogenetics, number of BM blasts (>26%), MDS overt AML and CB as stem cell source were significantly associated with worse prognosis (p = .03, p = .01, p = .02 and p < .001, respectively). In addition, based on a landmark analysis at 6 months post-transplant, the five-year Kaplan-Meier estimates of OS in patients with and without prior history of chronic GVHD were 64% and 17% (p = .022), respectively.

**Conclusion:**

Graft-versus-leukemia effects possibly mediated by chronic GVHD may have played a crucial role in long-term survival in, or cure of active leukemia.

## Introduction

Patients with primary refractory or refractory relapsed acute leukemia have an extremely poor prognosis. It has been generally recognized that few cases with primary refractory or refractory relapsed acute leukemia can be cured using conventional chemotherapy alone [1]. While allogeneic hematopoietic cell transplantation (allo-HCT) has the potential to cure even active leukemia, it has not been determined what subgroup can receive a long-term benefit from it.

Several retrospective studies have reported the prognostic factors for allo-HCT in patients not in remission at allo-HCT including untreated first relapse cases [2-8]. However, the factors contributing to long-term survival have not been established because the follow-up periods of these studies were not long enough at less than five years. Importantly, it can be assumed that patients who survive for more than five years without leukemia relapse are most likely cured. Only one large-scale retrospective study has examined long-term outcomes for more than five years following allo-HCT in adult patients with acute leukemia not in remission [9]. This study showed that several pre-transplant variables including complete remission duration, type of donor, disease burden, performance status, age and cytogenetics affected survival. However, whether post-transplant variables such as acute or chronic graft-versus-host disease (GVHD) influenced the post-HCT prognosis was not assessed. To our knowledge, no studies have investigated pre- and/or post-transplant factors which are associated with long-term survival exclusively in adult patients with active leukemia at allo-HCT. Therefore, we comprehensively evaluated the pre- and post-transplant factors which contribute to long-term survival of more than five years in patients with leukemia not in remission at allo-HCT.

## Patients and methods

Between January 1999 and July 2009, 42 consecutive patients (24 males and 18 females) with leukemia not in remission, aged 15 to 67 years (median age: 39 years), underwent allo-HCT at our institution. Patients with de novo acute myeloid leukemia (AML; n = 17), acute lymphoblastic leukemia (ALL; n = 12), chronic myeloid leukemia in accelerated phase (CML-AP; n = 2), myelodysplastic syndrome (MDS) overt AML (n = 10) and plasma cell leukemia (n = 1) were included. High-risk AML was defined according to the Eastern Cooperative Oncology Group/Southwest Oncology Group classification as having poor-risk cytogenetics (5/del[5q], 7/del[7q], inv[3q], abn11q, 20q or 21q, del[9q], t[6;9], t[9;22], abn17p, and complex karyotype defined as three or more abnormalities) [10]. High-risk ALL was defined as having poor-risk cytogenetics with either t(4:11), t(9;22), t(8;14), hypodiploidy or near triploidy, or more than five cytogenetic abnormalities [11]. Of study subjects with acute leukemia, cytogenetic abnormalities were intermediate (n = 17, 44%) or poor (n = 22, 56%). Seven patients were primary refractory to induction chemotherapy. The other patients relapsed after conventional chemotherapy (n = 23) or the first or the second HCT (n = 9). The median number of blast cells in bone marrow (BM) was 26.0% (range; 0.2-100) before the start of chemotherapy for allo-HCT. Six patients had leukemic involvement of the central nervous system (CNS). Stem cell sources were related BM (n = 3, 7%), related peripheral blood (PB) (n = 13, 31%), unrelated BM (n = 20, 48%) and unrelated cord blood (CB) (n = 6, 14%). Standard serologic typing was used for human leukocyte antigen (HLA) -A, B and DRB1. Thirty-one pairs were matched for HLA-A, B and DRB1 antigens. Three patients were mismatched for one HLA antigen (two at HLA-A, one at HLA-B), and seven were mismatched for two (two at HLA-A and B, five (all CB) at HLA-B and DRB1). The remaining one patient was mismatched for all three antigens (haploidentical). We classified conditioning regimens into four categories. Standard conditioning (n = 12) comprised a busulfan-based or total body irradiation (TBI)-based (12Gy) regimen. Busulfan was given as a total of 16 mg/kg orally or equivalent dose, 12.8 mg/kg intravenously (i.v.). Intensified conditioning (n = 9) consisted of additional cytoreductive chemotherapy in the three weeks before conditioning, followed by standard conditioning. Of the 21 patients receiving standard or intensified conditioning, 13 patients received the TBI-based regimen. Reduced-intensity conditioning (n = 21) comprised a fludarabine-based (n = 20) and cladribine-based regimen (n = 1). Fludarabine was given as 25-35 mg/m^2 ^i.v. on five or six consecutive days. Of the 21 patients receiving reduced-intensity conditioning, 14 patients received cytoreductive chemotherapy in the three weeks before conditioning. Prophylaxis for acute GVHD was a calcineurin inhibitor alone (n = 5), calcineurin inhibitor plus short-term methotrexate (n = 32), calcineurin inhibitor plus mycophenolate mofetil (n = 2), or none (n = 3). The calcineurin inhibitor included cyclosporine administered to 33 patients and tacrolimus to six patients.

### End points

The absence of post-transplant remission in some patients biased the calculation of relapse rate, nonrelapse mortality (NRM) and leukemia-free survival (LFS). Therefore, we set five-year overall survival (OS) as the primary end point. OS was defined as time from the date of last transplantation to the date of death or last follow-up. LFS was defined as time from the date of last transplantation to the date of disease relapse, death during remission or last follow-up. NRM was defined as a death not related to disease. Neutrophil recovery was defined as an absolute neutrophil count of at least 500 cells/mm^3 ^for three consecutive time points. Platelet recovery was defined as a count of at least 20 000 platelets/mm^3 ^without transfusion support. Acute GVHD (aGVHD) was defined in accordance with standard criteria [12]. Chronic GVHD (cGVHD) was evaluated in patients surviving for more than 100 days after allo-HCT and was classified into limited or extensive type [13].

### Statistical analysis

If the disease for which the patient underwent transplantation was present at the time of death or found at autopsy, we defined disease relapse/progression as the primary cause of death. Unadjusted survival probabilities were estimated using the Kaplan and Meier method and compared using the log-rank tests. Cumulative incidence curves were used in a competing-risks model to calculate the probability of aGVHD, cGVHD and NRM [14]. For neutrophil and platelet recovery, death before neutrophil or platelet recovery was the competing event; for GVHD, death without GVHD and relapse were the competing events; and, for NRM, relapse was the competing event. In order to examine the impact of cGVHD on survival, we performed a landmark analysis, which divided patients according to their prior history of cGVHD at 6 months post-transplant [15]. We excluded from landmark analysis patients who died or relapsed less than 6 months after transplant, and did not use the information on whether or not patients developed cGVHD 6 months after transplant. Multivariable analysis of prognostic factors for the primary outcome could not be conducted due to lack of statistical power. Instead, we performed a landmark analysis, which divided patients according to the significant pre-transplant factors and their prior history of cGVHD at 6 months post-transplant. All P values were 2-tailed and considered statistically significant if the values were less than 0.05. All statistical analyses were performed using the PASW Statistics17.0 (SPSS Inc, Chicago, IL, USA) and the statistical software environment R, version 2.9.1.

## Results

The baseline characteristics of the patients are shown in Table 1.

**Table 1:** Baseline characteristics of study participants

Variable	n (%)	Median (Range)
Male sex	24 (57.1)	
Diagnosis		
de novo AML	17 (40.5)	
ALL	12 (28.6)	
CML-AP	2 (4.8)	
MDS overt AML	10 (23.8)	
PCL	1 (2.4)	
Cytogenetics		
Intermediate	17	
Poor	22	
ECOG PS		
0	2 (4.8)	
1	25 (59.5)	
2	7 (16.7)	
3	8 (19.0)	
Status at allo-HCT		
Primary refractory/Refractory relapse/Untreated MDS overt AML	7/32/3	
No. chemo regimens prior allo-HCT		6 (0-18)
Time from diagnosis to allo-HCT (days)		319 (23-3738)
Marrow blasts at allo-HCT		26.0 (0.2-100)
Conditioning regimen		
Intensified	9 (21.4)	
Standard	12 (28.6)	
Reduced-intensity	7 (16.7)	
Reduced-intensity + cytoreductive chemotherapy	14 (33.3)	
GVHD prophylaxis		
None	3 (7.1)	
Calcineurin inhibitor alone	5 (11.9)	
Calcineurin inhibitor + sMTX	32 (76.2)	
Calcineurin inhibitor + MMF	2 (4.8)	
Donor (HLA-A, B and DRB1 antigens)		
Matched related PB/BM	10/2	
Mismatched related PB/BM	3/1	
Matched unrelated BM	19	
Mismatched unrelated BM	1	
Umbilical cord blood	6	

allo-HCT: allogeneic hematopoietic cell transplantation; HLA: human leukocyte antigen; sMTX: short-term methotrexate; MMF: mycophenolate motefil; BM: bone marrow; PB: peripheral blood.

### Engraftment

Neutrophil engraftment was achieved in 33 (79%) of 42 patients. The median time to neutrophil engraftment was 17 days (range, 9-32). In a total of four of 27 evaluable patients, a platelet count > 20 000/μl was not achieved. In the patients that achieved platelet counts of ≥ 20 000/μl, the median time to platelet engraftment was 33 days (range, 13-99). The cumulative probabilities of neutrophil and platelet engraftment were 79% and 55%, respectively.

### GVHD

Twenty-four of 42 patients developed aGVHD (eight grade I, nine grade II, five grade III, two grade IV). Twelve of 24 evaluable patients developed cGVHD (one limited, 11 extensive). At five years, the cumulative probabilities of aGVHD and cGVHD were 63% and 37%, respectively.

### NRM

A total of eight patients were alive at the time of this analysis, seven in complete remission (CR). The most common cause of death was disease relapse/progression. Causes of death were disease relapse/progression (n = 27), GVHD (n = 2), sinusoidal obstruction syndrome (SOS) (n = 3), Epstein-Barr virus associated post-transplant lymphoproliferative disorder (n = 1), and adenovirus infection (n = 1). Of six patients with CNS lesion, five died of disease relapse/progression (n = 3), GVHD (n = 1) and SOS (n = 1), and one was alive at last follow-up although another HCT was planned due to BM relapse post-transplant. At five years, the cumulative probability of NRM was 38%. Nine patients died before day 30, and 18 patients died within the first 100 days post-HCT.

### LFS and OS

A total of 22 of 33 evaluable patients attained a CR after the allo-HCT. The median follow-up of survivors was 85 months (range, 24-126 months). The five-year Kaplan-Meier estimates of LFS and OS were 17% and 19%, respectively.

### Univariable analysis

**Figure 1:**
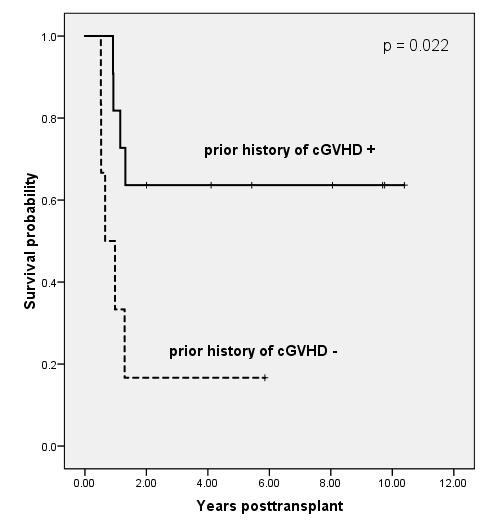
**Kaplan-Meier estimates of overall survival based on a landmark analysis at 6 months post-transplant, grouping patients according to prior history of cGVHD (p = .022)**. The 5-year survival rates of patients with and without prior history of cGVHD were 64% and 17%, respectively.

We analyzed the impact of pre- and post-transplant characteristics on OS after allo-HCT. The factors included age at transplant, sex, primary vs. secondary leukemia, cytogenetics at diagnosis, number of BM blasts, donor type, myeloablative vs. reduced-intensity conditioning, and presence or absence of acute and chronic GVHD. Results of univariable analysis for OS are summarized in Table 2. In the univariable analyses of the impact of pre-transplant variables on OS, poor-risk cytogenetics, number of BM blasts (>26%), MDS overt AML and CB as stem cell source were significantly associated with worse prognosis (p = .03, p = .01, p = .02 and p < .001, respectively). In addition, based on a landmark analysis at 6 months post-transplant, the five-year Kaplan-Meier estimates of OS in patients with and without prior history of cGVHD were 64% and 17% (p = .022) respectively (Figure 1).

**Table 2:** Univariable analysis of impact of pre-transplant variables on overall survival

Variable	Survival (% at 5 y)	Log rank P value
Age at allo-HCT		
< 40	28	0.055
≥ 40	6	
		
Diagnosis		
MDS overt AML	0	0.015
Others	25	
		
Cytogenetics		
intermediate	35	0.013
poor	5	
		
Marrow blasts at allo-HCT		
≤ 26	33	0.013
> 26	5	
		
Donor source		
Umbilical cord blood	0	<0.001
Others	22	
		
Conditioning		
Intensified	22	0.087
Standard	42	
Reduced-intensity	0	
Reduced-intensity + cytoreductive chemotherapy	7	

allo-HCT: allogeneic hematopoietic cell transplantation

### Bivariable analysis

**Figure 2:**
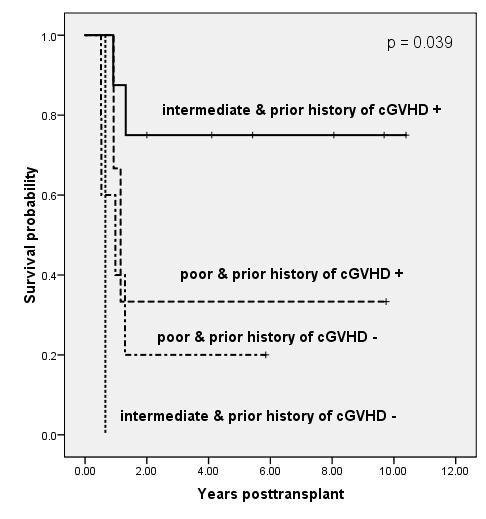
**Kaplan-Meier estimates of overall survival based on a landmark analysis at 6 months post-transplant, grouping patients according to cytogenetics and prior history of cGVHD (p = .039)**. The 5-year survival rates of patients with intermediate & prior history of cGVHD +, poor & prior history of cGVHD +, and poor & prior history of cGVHD - were 75%, 33%, and 20%, respectively.

**Figure 3:**
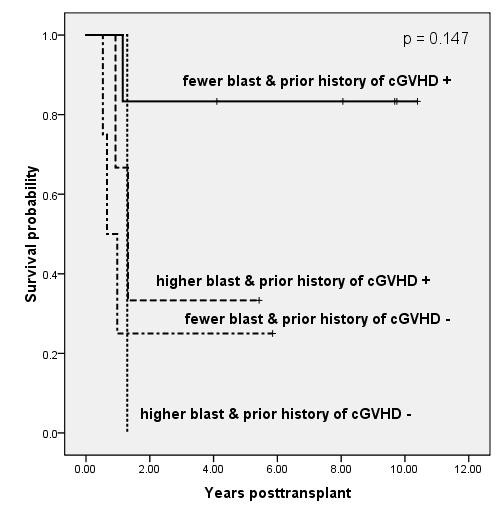
**Kaplan-Meier estimates of overall survival based on a landmark analysis at 6 months post-transplant, grouping patients according to percent marrow blast (≤ or > 26%) at baseline and prior history of cGVHD (p = .147)**. Patients with CNS lesion were not included in this analysis. The 5-year survival rates of patients with fewer blast & prior history of cGVHD +, higher blast & prior history of cGVHD +, and fewer blast & prior history of cGVHD - were 83%, 33%, and 25%, respectively.

**Figure 4:**
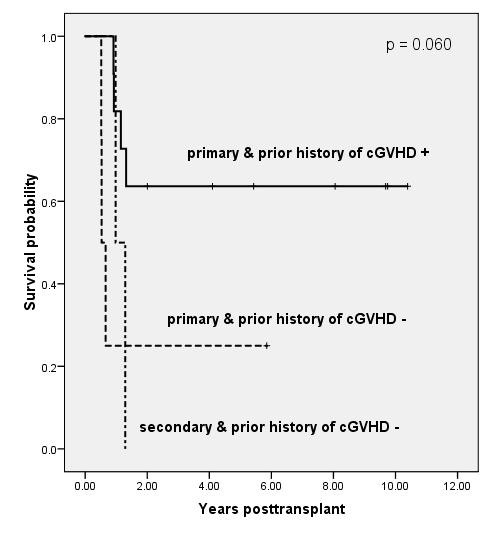
**Kaplan-Meier estimates of overall survival based on a landmark analysis at 6 months post-transplant, grouping patients according to primary or secondary leukemia and prior history of cGVHD (p = .060)**. The 5-year survival rates of patients with primary & prior history of cGVHD + and primary & prior history of cGVHD - were 64% and 25%, respectively.

We performed the landmark analyses at 6 months post-transplant, which classified patients according to significant pre-transplant factors including poor-risk cytogenetics, number of BM blasts, or secondary leukemia and their prior history of cGVHD at 6 months post-transplant. Results of bivariable analysis for OS are shown in Figure 2, Figure 3 and Figure 4. The groups of patients with intermediate cytogenetics, marrow blast ≤ 26% or primary leukemia, who developed cGVHD less than 6 months after transplant, showed significantly or borderline significantly higher survival rates than those in the other groups (p = .039, p = .147, and p = .060, respectively). The five-year Kaplan-Meier estimates of OS in the patients with intermediate cytogenetics, marrow blast ≤ 26% or primary leukemia in addition to prior history of cGVHD were 75%, 83%, and 64%, respectively.

## Discussion

Our data showed that allo-HCT resulted in long-term disease remission and an eventual cure of active leukemia in a subset of de novo AML or ALL patients with marrow blast ≤ 26% and without poor-risk cytogenetics, possibly by graft-versus-leukemia (GVL) effects mediated through cGVHD.

A retrospective study with a large cohort using data reported to the Center for International Blood and Marrow Transplant Research demonstrated that pre-transplant variables delineated subgroups with different long-term allo-HCT outcomes in adult patients with acute leukemia not in remission [9]. However, they did not address the effect of cGVHD on survival. Baron et al. have reported that extensive cGVHD was associated with decreased risk of progression or relapse in patients with AML or MDS in complete remission at the time of nonmyeloablative HCT [16]. However, it remains unclear whether cGVHD is associated with long-term disease control in patients who have active leukemia at transplant. The results of the current study showed that GVL effects mediated by cGVHD may play a crucial role in long-term survival in or a cure of active leukemia, especially in patients without poor-risk cytogenetics. Further study on the possible relationship between cGVHD and GVL effects would be very helpful in the management of immunosuppressive treatment.

For patients who were ineligible for myeloablative conditioning due to comorbidities coupled with rapidly progressive leukemia, we administered sequential cytoreductive chemotherapy, followed by reduced-intensity conditioning for allo-HCT in order to reduce toxicity and obtain sufficient anti-leukemic efficacy. The utility of the combination of sequential cytoreductive chemotherapy and reduced-intensity conditioning for allo-HCT was previously reported [17]. Our results did not show that this sequential regimen had an advantage in controlling active leukemia. However, we speculated that effective tumor reduction by individual chemotherapy and/or conditioning for allo-HCT to control disease until cGVHD subsequently occurred might also be important, particularly in rapidly proliferating leukemia. In contrast, intensive conditioning did not appear to be essential in relatively indolent leukemia, even with non-remission.

Based on our results, CB might be unsuitable as a source of stem cells for treatment of active leukemia at the time of allo-HCT. However, most patients receiving CBT could not wait for an unrelated donor search because their disease tended to be aggressive compared with those in the unrelated BM group. Thus, it is difficult to arrive at any conclusions about the best stem cell source for allo-HCT in patients in non-remission status based solely on our results.

Our study has several limitations. The results might be affected by an underlying selection bias due to the nature of retrospective data. Also, our study was limited by the small number of patients, the heterogeneity of the disease, the transplant procedure and the stem cell source. However, the major strengths of our study were that the follow-up period was sufficient with more than 5 years and the impact of cGVHD as well as pre-transplant factors on long-term survival were analyzed exclusively for subjects with active leukemia.

## Conclusion

These data show that allo-HCT has the potential to cure active leukemia possibly via cGVHD, particularly in patients with favorable factors even when in non-remission. Further research is warranted to explore the essential factors contributing to the success of allo-HCT such as intensity of conditioning, and GVL effects mediated through cGVHD.

## Competing interests

The authors declare that they have no competing interests.

## Authors' contributions

HK and HN designed the study and wrote the paper; HK analyzed results and created the figures; MH designed the research; M Nakamae and YU reviewed the patients' medical records and cleaned the data; MO reviewed the pathological specimens in this study; and KH, TN, MM, YH, M Nishimoto, AH, EI, AI, MY, MB, HO, RA, MA, YT, KK, TY reviewed the results. All authors have read and approved the final manuscript.
